# ArcTEX—a novel clinical data enrichment pipeline to support real-world evidence oncology studies

**DOI:** 10.3389/fdgth.2025.1561358

**Published:** 2025-05-09

**Authors:** Keiran Tait, Joseph Cronin, Olivia Wiper, Jamie Wallis, Jim Davies, Robert Dürichen

**Affiliations:** ^1^ Arcturis Data, Kidlington, United Kingdom; ^2^ Department of Computer Science, University of Oxford, Oxford, United Kingdom

**Keywords:** natural language processing, data enrichment, real-world data, electronic health records, oncology

## Abstract

Data stored within electronic health records (EHRs) offer a valuable source of information for real-world evidence (RWE) studies in oncology. However, many key clinical features are only available within unstructured notes. We present ArcTEX, a novel data enrichment pipeline developed to extract oncological features from NHS unstructured clinical notes with high accuracy, even in resource-constrained environments where availability of GPUs might be limited. By design, the predicted outcomes of ArcTEX are free of patient-identifiable information, making this pipeline ideally suited for use in Trust environments. We compare our pipeline to existing discriminative and generative models, demonstrating its superiority over approaches such as Llama3/3.1/3.2 and other BERT based models, with a mean accuracy of 98.67% for several essential clinical features in endometrial and breast cancer. Additionally, we show that as few as 50 annotated training examples are needed to adapt the model to a different oncology area, such as lung cancer, with a different set of priority clinical features, achieving a comparable mean accuracy of 95% on average.

## 1 Introduction

Electronic health records (EHR) represent a vast and increasingly utilized source of information for real-world evidence (RWE) generation. Examples of such applications include retrospective cohort studies, comparative effectiveness studies, and the creation of external control arms (1). In oncology, molecular profiling is increasingly being used to guide patient management, enabling more personalised treatment approaches. For example, molecular profiling of endometrial cancer has revealed at least four distinct subtypes of disease, each requiring different approaches to optimal clinical management (2). However, many of these key biomarkers are often recorded in unstructured clinical reports rather than structured data, making it challenging to incorporate them into real-world evidence studies. In general, it is estimated that at least half of all information is stored in unstructured form (3). Further automatic processing of this text remains challenging because of the unique language and idioms used by clinicians (4). This poses significant challenges for evidence generation as key insights into symptom profiles, clinical markers, response to treatments, or other clinically relevant areas are not directly accessible.

The manual extraction of this information is time consuming, binds capacity of clinical experts and often not practically possible to do at scale (4). Various natural language processing (NLP) methods have been developed to tackle this problem ranging from simple rule-based methods, N-grams to more complex machine learning approaches such as support vector machines, recurrent neural networks or pretrained text embeddings like Glove or word2vec (5). In 2019, the field was massively impacted by the development of transformer-based machine learning methods such as BERT (6) and its domain specific adaptations such as BioBERT (7). These models can be considered as discriminative models (DM) as they only consist of an encoder structure and are not able to generate new text. Recently these techniques have been further developed and resulted in the development of large language models (LLMs) such as Llama by Meta (8) which have shown promising results. LLMs are often defined as models with over 1 billion parameters and are often generative models (GMs), as they consist of an encoder-decoder or decoder only architecture, which can be used to generate new text, for instance to answer questions or summarize text.

Beside these technical advancements, the problem of extraction of clinical terms, also referred to as name entity recognition (NER), is still not solved as demonstrated by Abdul et al. (9). The authors released a leaderboard consisting of 4 publicly available clinical NER datasets which contain clinical conditions, procedures, medications and laboratory test names. They benchmarked various state of the art models such as Llama, Open AI GPT4o or Mixtral. The best performing model achieved an average F1-score of only 77.1%, indicating that it is still challenging for a single model to extract a wide range of clinical terminologies accurately.

A second finding is that, even though the generative capabilities of LLMs are impressive (e.g., text summarization, question-answering systems), LLMs are not always superior compared to alternative DMs such as BERT based models. Chen et al. showed that BERT models have a higher accuracy compared to GPT3.5 and GPT4 on tasks like clinical NER and relation extraction (RE) (10). A similar conclusion was reported by Zhu et al. in their paper “*Is larger always better?”* (11) and Chen et al. (12).

From an application point of view, multiple technical and legal aspects need to be considered when NLP algorithms are used to extract information from clinical notes. First, free-text clinical reports often contain both sensitive medical information as well as personally identifiable information (PII). In the case of a data breach, this could pose a major risk to both the individuals whose data has been leaked, as well as to the healthcare providers who held the data. As an example, many NHS Trusts (The organizational units that encompass regional/local NHS services such as hospitals within the UK) don't allow external partners direct access to the free text reports. In some cases, deidentification protocols can be used to redact PII from free-text reports to give external users access to the reports. These methods, however, are often overzealous to ensure total PII redaction, and can result in data loss through misidentification of text for redaction. An alternative approach would be to send only the NLP algorithm to the data instead of providing direct access to the data. In this “*blind case*” scenario, it needs to be guaranteed that the outcome of the NLP algorithm can be trusted and is free of any PII.

Second, many UK Trust environments have only limited hardware resources available. Trust environments are often resource constrained due to lack of requirement for GPU based computer resources. Additionally, the usage of web-based APIs to use LLMs is challenging due to risk of PII leaks as discussed previously. Additionally, some Trusts keep their EHR data in an environment without any, or only limited, internet access to increase data security, which eliminates the possibility of using of web-API based LLMs. The general availability of on-site GPUs required to execute some of the larger LLMs, therefore cannot be assumed. Smaller models, such as BERT based models, with less than 100–300 million parameters can still be executed in a resource constrained environment with only CPUs.

In this paper, we present a novel data enrichment model ArcTEX, which stands for **Arc**turis **T**ext **E**nrichement and e**X**traction. The approach was developed to extract oncology specific clinical features such as specific biomarkers relevant for endometrial or breast cancer with high accuracy to support RWE studies using UK based clinical notes. Further, the model is designed to operate within a resource constrained clinical environment, such as in hospitals or other clinical settings. The model output is free of PII by design, and it provides confidence scores which can be used to increase accuracy of downstream analysis.

The contributions of the paper are threefold:

• *Model architecture:* Detailed presentation of the model architecture of ArcTEX and its subcomponents,
• *Comparison:* Evaluation of ArcTEX and baseline NLP models (both discriminative and generative) on 18 oncology related clinical features with respect to accuracy and computation time (experiment 1), and
• *Adaptability:* Investigation on amount of training samples required to achieve a high accuracy for new unseen clinical features for ArcTEX and selected baseline models (experiment 2).

## 2 Materials and methods

### 2.1 Dataset and annotation

This study took 3,903 individual reports from a wider dataset of 77,693 fully-anonymised free-text pathology reports provided by Oxford University Hospital via the Thames Valley and Surrey Secure Data Environment (min-max number of words per report: 7-3213 words; mean number of words per report: 339.9). This dataset consists of a population of oncology patients, with at least one diagnosis of lung, pancreatic, renal, breast, ovarian, liver, or endometrial cancer. The dataset was generated by applying a PII reduction algorithm on the original reports. Potential PII was replaced in the report with “*[redacted]”*.

Annotations were performed with a focus on endometrial and breast cancer related clinical features. Additionally, a clinical feature from a different oncology area than what the focus of the dataset was, namely hematological malignancies, was annotated. This feature was chosen based on low relative frequency and data type (i.e., quantitative vs. qualitative) to provide a wider range of types of features to test the models against. In total 18 clinical features were annotated:

• Endometrial cancer: FIGO stage, grade, p53, MMR, MLH1, MSH2, MSH6, PMS2, myometrial invasion, and lymphovascular invasion
• Breast cancer: HER2, ER, and PR
• TNM staging: T, N, and M stages and edition used
• Blast Cell Percentage

In total, 6,514 manual annotations were created. The annotations could be split into two main groups. Firstly, those where presence of a given feature can be positively identified (e.g., *HER2: positive/negative/not mentioned*; *FIGO stage: 2a/2b/3a*…), otherwise known as a positively identifiable answer. The second group consisted of annotation where there was an absence of positively identifiable clinical feature (e.g., “…p53 has been requested…”, or reports missing the feature entirely) otherwise known as an “impossible answer”. In reports where a feature was mentioned multiple times, for example directly quoting a previous report and amending the result with more up to date results, the most clinically severe result was annotated. The manually annotated reports were then partitioned into a test set consisting of 100 reports, and the remaining reports made up the training set, containing 4,714 reports.

Two annotators performed annotations over the dataset, with Annotator 1 annotating the entire dataset and Annotator 2 annotating 10% of the dataset to validate the ground truth. Annotator 1 has a background in Medical Visualisation and Human Anatomy, and has worked previously with histopathological methods in both academic and teaching settings. Annotator 2 has a background in medicine, health data science and cancer biology. She practiced as a junior doctor for 5 years, with experience in interpreting pathology reports.

The annotations described above were used for both experiments explored in this manuscript. Experiment 1 focused on the comparison of each of the interrogated models ability to extract the statuses of each of the 18 clinical features mentioned above. For experiment 2, the adaptability of the LLMs was to be tested by exploring unseen clinical features in a different disease area, in this case lung cancer. Reports featuring an additional 5 clinical features associated with lung cancer were therefore annotated; namely anaplastic lymphoma kinase (ALK), chromogranin (CgA), epidermal growth factor receptor (EGFR), synaptophysin, and thyroid transcription factor 1 (TTF1). A total of 200 reports with identifiable features (e.g., *positive, negative*) and 100 with absence of clinical features [e.g *not mentioned, (redacted)*] were annotated.

### 2.2 ArcTEX model

**Figure 1:**
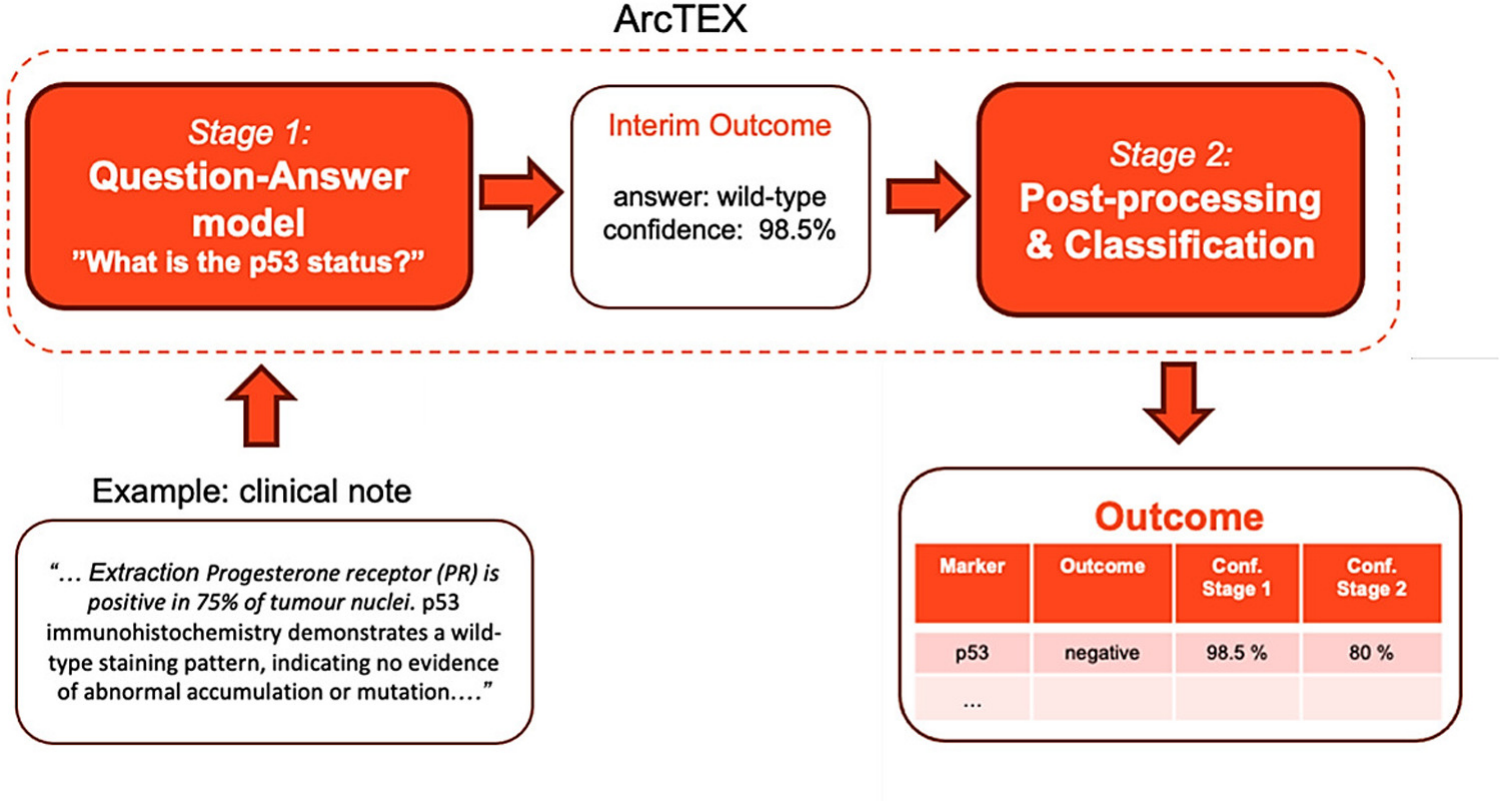
Schematic illustration of the ArcTEX pipeline which uses a 2-stage question-answering approach, illustrated on the example extraction of p53 clinical feature.

The ArcTEX pipeline is made up of two main stages: a question and answering (QA) stage and a postprocessing stage (Figure 1) In the QA stage (stage 1), a free-text clinical report (context) is fed into a question-answering model along with a targeted clinical question, e.g., “*What is the p53 status?”.* The model then outputs the predicted answer along with a confidence score, e.g., [“*wild-type*”, *0.985*]. The base model utilised in this stage is a BioBERT (7) QA model, available from HuggingFace^1^. This BioBERT model is a BERT based model pre-trained on a large corpus of biomedical text and the Stanford Question Answering Dataset [SQuAD (13)]. In contrast to generative models, this model only predicts the start and end token, which is most likely to answer the question, ensuring that no hallucinations will be generated. The model can handle text where the question is unable to be answered based on available data (impossible answers), and will return an empty string if it does not believe the answer is contained within the context. During initial testing, the BioBERT model was found to be the most accurate model compared to other common BERT based QA models [RoBERTa (14) and PubMedBERT (15)]. The BioBERT model was further finetuned on 4,714 annotated free-text reports obtained from Oxford University Hospital covering descriptions of 18 distinct clinical features, resulting in the “ArcTEX” model.

In the postprocessing stage (stage 2), the predicted answers from the QA model are classified into predefined, clinically relevant categories specific to a given biomarker, e.g., *p53: positive/negative*. Note that these classes will differ between biomarkers depending on the values the biomarker can take, but every biomarker will have an additional class “*not present”* that indicates there is no mention of the biomarker value present in the report. See Table 1 for a full list of biomarker classes. As an initial step, blank answers and low-confidence answers (<10%) from the QA stage are automatically set to the “*not present”* class. The remaining answers are fed into a SetFit (16) classifier, a prompt-free framework for few-shot fine-tuning of sentence transformers capable of achieving high accuracy from a small number of labelled training examples. This was done as an improvement upon the best performing model in initial tests which used a cosine similarity approach. For each biomarker, this required training a sentence transformer on 5–10 annotated examples per class to capture the variance in how biomarker values were written in different reports. Mpnet-v2^2^ was used as a sentence transformer. For each report, the ArcTEX pipeline then outputs a predicted clinical category along with the confidence scores of the QA stage and the SetFit classifier (see Figure 1). This ensures that no PII can be leaked from the outputs and allows the user to tweak the outputs by applying their own custom thresholds to the model confidence scores.

**Table 1:** List of possible classes for each clinical feature.

Clinical feature	Classes
ALK	Positive, Negative, Equivocal
Blast cell Percentage	[0–100%], No Excess, An Excess
CgA	Positive, Negative
EGFR	Positive, Negative
ER	Positive, Negative, [0–8]
FIGO	1,1a,1b,1c,2,2a,2b,3,3a,3b,3c,4,4a,4b
Grade	1,2,3,4
Her2	Positive, Equivocal, Negative, 1, 2, 3
Lymphovascular Invasion	Present, Absent
Myometrial Invasion	Present, Absent
MMR	Intact, Deficient
MLH1	Present, Absent
MSH2	Present, Absent
MSH6	Present, Absent
PMS2	Present, Absent
PR	Positive, Negative, [0–8]
P53	Positive, Negative
TNM staging T	0,1,1a,1b,1c,2,3,3a,3b,4,4a,4b
TNM staging N	X,0,1,1a,1b,2,2a,2b,2c,3,3a,3b
TNM staging M	X,0,1,1a,1b,1c
TNM staging Edition	5,7,8
Synaptophysin	Positive, Negative

Where numerical features are present, a larger range is represented in square brackets.

### 2.3 Baseline models

To evaluate the ArcTEX model, the two stage ArcTEX pipeline was used to test a selection of DMs: RoBERTa and BioBERT, as well as larger GMs: Llama3-8B-Instruct, Llama3.1-8B-Instruct, Llama3.2-3B-Instruct and Llama3.2-1B-Instruct against the ArcTEX model itself. Llama models with a higher number of parameters were excluded from the evaluation as they could not be executed in a resource constrained environment (an environment ideally without a GPU). To allow for a fairer comparison, all models were finetuned using the same dataset that ArcTEX was trained on. The finetuned models were abbreviated as *model_name (ft).* For the DMs, the finetuning was performed identically given the shared training schema used between them. The GMs were also trained in a supervised manner, however, due to the significant size of these models they were trained using Quantized Low-Rank Adaptation (QLoRA) (17, 18) on a GPU machine. A description of each model evaluated can be found in Table 2.

**Table 2:** Description of models evaluated.

Model name	Description
ArcTEX	Huggingface BioBERT finetuned on 4,714 free-text pathology reports, using a SetFit classifier
BioBERT	Huggingface BioBERT model, using a cosine similarity classifier
BioBERT (ft)	Huggingface BioBERT finetuned on 4,714 free-text pathology reports, using a cosine similarity classifier
RoBERTa	Huggingface RoBERTa model, using a cosine similarity classifier
RoBERTa (ft)	Huggingface RoBERTa finetuned on 4,714 free-text pathology reports, using a cosine similarity classifier
RoBERTa (ft) + SetFit	Huggingface RoBERTa finetuned on 4,714 free-text pathology reports, using a SetFit classifier
Llama-3 8B	Huggingface Llama-3 8 billion parameter model with cosine similarity classifier
Llama-3 8B (ft)	Huggingface Llama-3 8 billion parameter model finetuned on 4,714 free-text pathology reports, with cosine similarity classifier
Llama-3.1 8B	Huggingface Llama-3 8 billion parameter model with cosine similarity classifier
Llama-3.1 8B (ft)	Huggingface Llama-3 8 billion parameter model finetuned on 4,714 free-text pathology reports, with cosine similarity classifier
Llama-3.2 1B	Huggingface Llama-3 8 billion parameter model with cosine similarity classifier
Llama-3.2 1B (ft)	Huggingface Llama-3 8 billion parameter model finetuned on 4,714 free-text pathology reports, with cosine similarity classifier
Llama-3.2 3B	Huggingface Llama-3 8 billion parameter model with cosine similarity classifier
Llama-3.2 3B (ft)	Huggingface Llama-3 8 billion parameter model finetuned on 4,714 free-text pathology reports, with cosine similarity classifier

**Figure 2:**
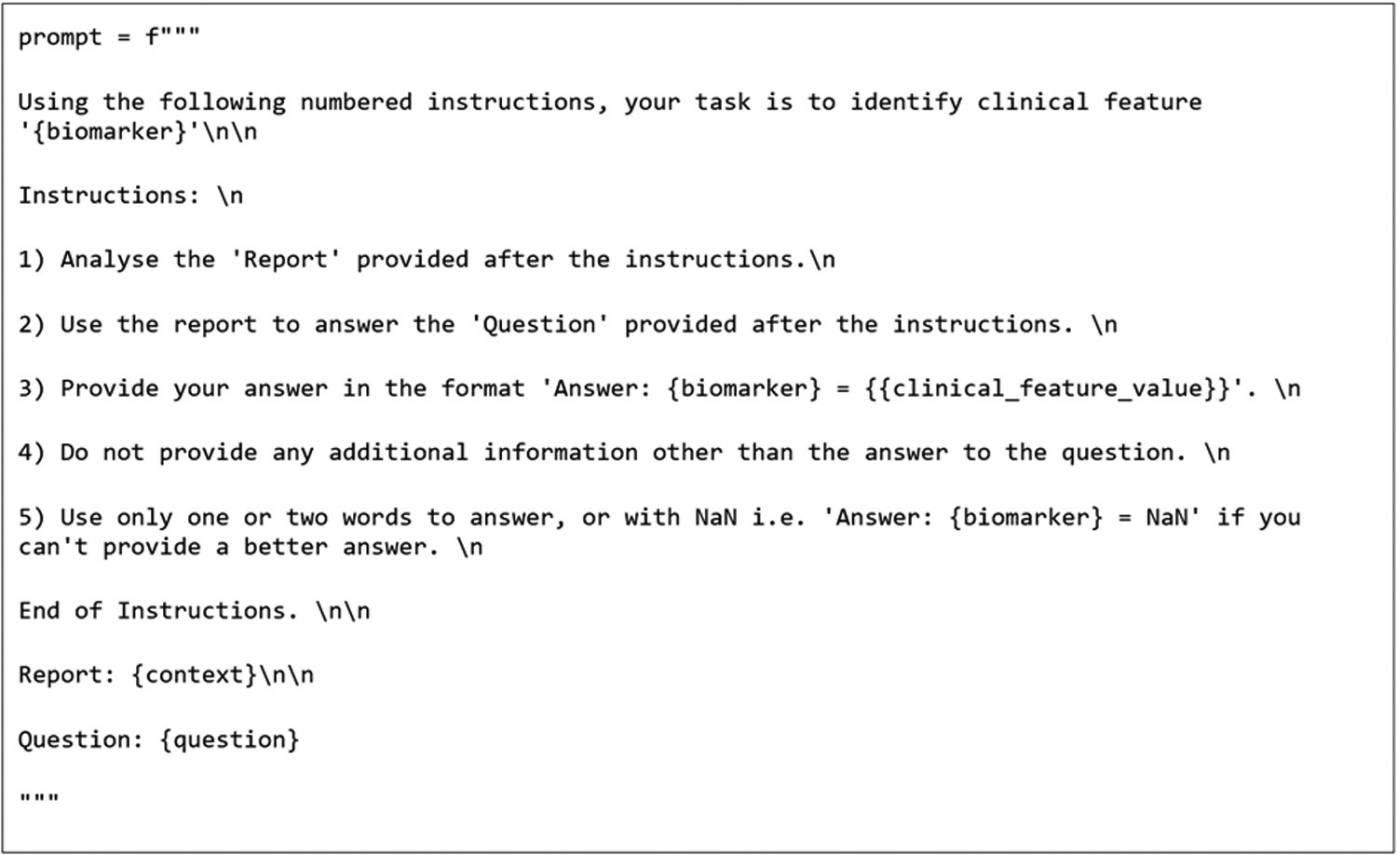
Prompt template used for question answering pipeline.

**Figure 3:**
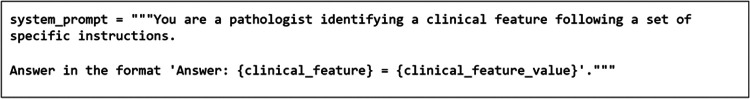
System prompt used for directing model to perform task in a particular way.

As the GMs require prompting, a role-based instructional approach was adopted. The prompt format that returned the reported results used both a “system prompt” and an “instructional prompt”. This method was demonstrated to be an effective prompting method previously (19). An example of the prompts used can be seen in Figures 2, 3. The bracketed question, context, and biomarker fields were filled with specific data relevant to each clinical feature and on a report-by-report basis. Additional special tokens were also added to the prompts as described by their model cards (20, 21).

To test the initial baseline models, the postprocessing stage was simplified to handle the variation in GM output. Firstly, regex was applied to extract the text following the biomarker string as per the prompt template in Figure 2. The extracted text was then classified into categories by computing the cosine similarities between the text embeddings and the embeddings of the same annotated training data used to train the SetFit classifiers. These embeddings were also generated using the Mpnet-v2^2^ sentence transformer. The baseline models were then finetuned and evaluated again in the same manner using cosine similarity. Additionally, RoBERTa was run with the SetFit classifier to compare it to ArcTEX. The GMs were not run with the SetFit classifier due to the way that the GMs generate their response. GMs possess the ability to generate text to respond to a question, and therefore do not rely on the actual text content to generate their response. Because of this, the additional time and resources associated with running and using a SetFit classifier over an approach like cosine similarity are unjustified. In comparison, because DMs directly lift text snippets from the queried text, they are sensitive to variability in spelling (including errors) or in reporting styles.

### 2.4 Experimental setup

#### 2.4.1 Experiment 1: model comparison

The objective of the first experiment is to evaluate the performance of the ArcTEX model compared to both types of baseline models—DMs and GMs. All models were evaluated in a base version (without finetuning) and finetuned versions using the two stage ArcTEX pipeline. The test set consisted of 100 reports for each of 18 clinical features and was excluded from any finetuning data used to train the finetuned models to ensure no overlap between test and training sets. For each of the test sets, 50 reports that contained a positively identifiable reference to the clinical feature (e.g., “*positive”, “negative”, “wild-type”, “3/8”, “+”*) and 50 that contained either no reference or a non-positively identifiable reference to the feature [e.g., “*[redacted]”, “…{feature} is requested…”*]. The 18 features annotated for were: FIGO stage, grade, p53, MMR, MLH1, MSH2, MSH6, PMS2, myometrial invasion, and lymphovascular invasion, HER2, ER, and PR, TNM stage (T, N, and M stages as separate features), TNM staging edition, and blast cell percentage. The accuracy was calculated as the percent of correctly identified and classified outputs as compared to the human annotated ground truth. The average accuracy across the above features was calculated per model, with the standard deviation around the average calculated based off the variation in results per feature. 95% Confidence Intervals (95% CI) were also calculated and are enclosed in square brackets beside the average scores. In addition to accuracy, F1-scores were also calculated with 95% CI and standard deviations, along with the average precision and recall as well as required runtime on the same hardware system.

#### 2.4.2 Experiment 2: adaptability

The objective of this experiment is to investigate how adaptable ArcTEX is compared to finetuned RoBERTa, BioBERT, and Llama 3.1-8B models for extracting new oncological features from a different oncological area. Therefore, the models were tested to extract lung cancer specific features namely: anaplastic lymphoma kinase (ALK), chromogranin (CgA), epidermal growth factor receptor (EGFR), synaptophysin, and thyroid transcription factor 1 (TTF1). For each of these five features, a test set of 50 examples with positively identified clinical markers, and 50 examples with negatively identified clinical markers was generated from the pool of ground truth annotations (200 annotated reports for each clinical feature). These remained consistent in the testing of the model. The remaining 100 samples were set aside to form the training sets. For the analysis, the experiment was repeated 5 times over a set of 5 permutations of the afore mentioned test/training set split. For each permutation, the training set was further subdivided into varying sizes of 5, 10, 20, 50, and 100 reports using random sampling. The average accuracy across all five features were computed depending on the size of the training set to investigate the amount of training data required to reach an accuracy plateau.

### 2.5 Technical setup

The computations were performed on AWS cloud platform using a g6.2xlarge instance^3^, with 32 GiB memory, a single NVIDIA L4 GPU and 8 vCPUs.

## 3 Results

### 3.1 Interrater variability

A subset of 10% of the groundtruth was annotated by a secondary annotator to evaluate interrater variability. Agreement was measured with Cohen's Kappa coefficient, and was found to be 0.962 on average, with a 95% CI of 0.026 indicating high agreement between raters.

### 3.2 Experiment 1: model comparison

**Figure 4:**
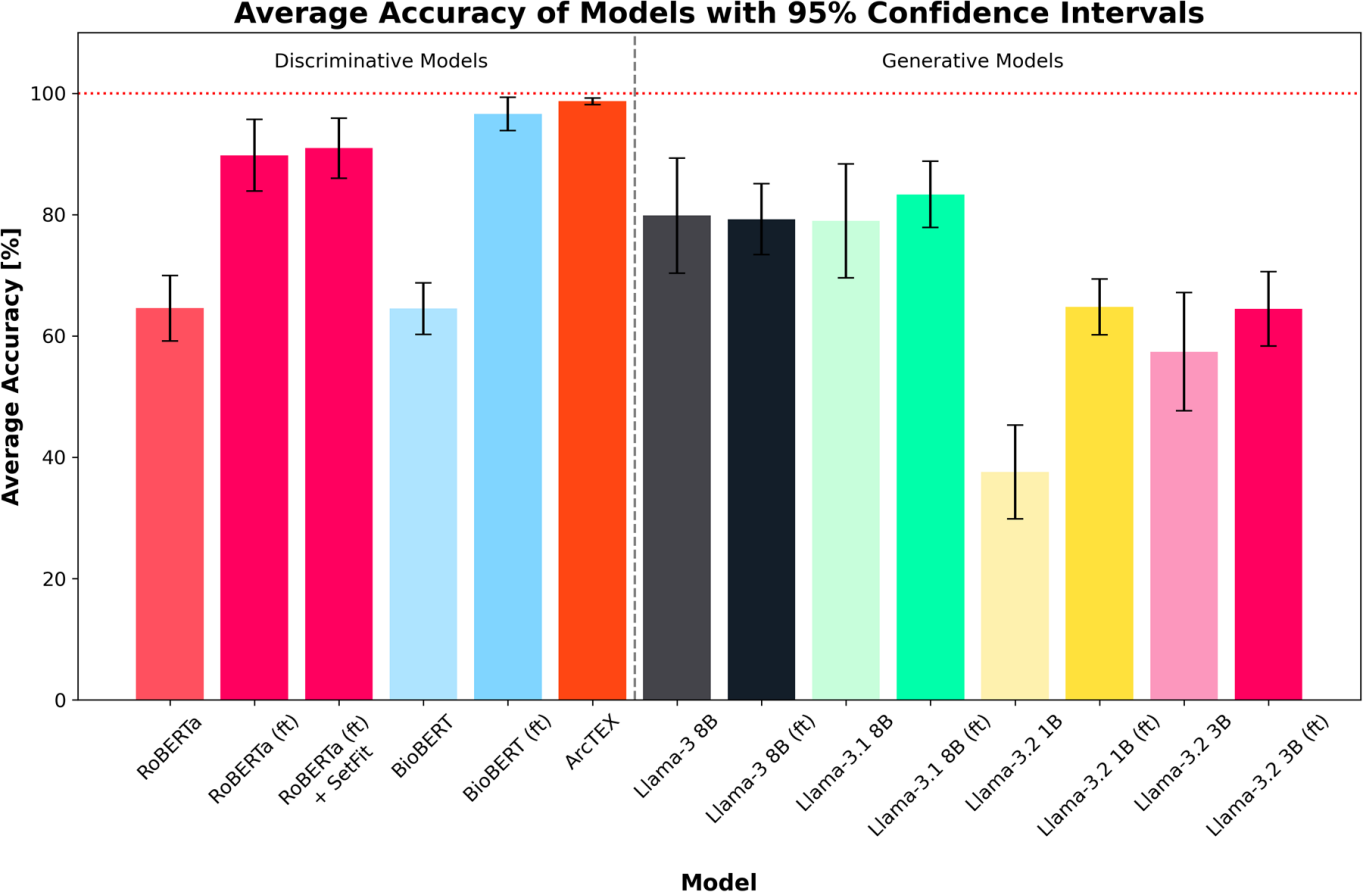
Average accuracies for each model across all test sets. Error bars show 95% CI. Finetuned models are marked by (ft). ArcTEX shows the highest performance overall with the lowest confidence interval range. In general, the base GMs perform comparably to better than the baseline DMs, however with finetuning the DMs outperform all GMs.

**Figure 5:**
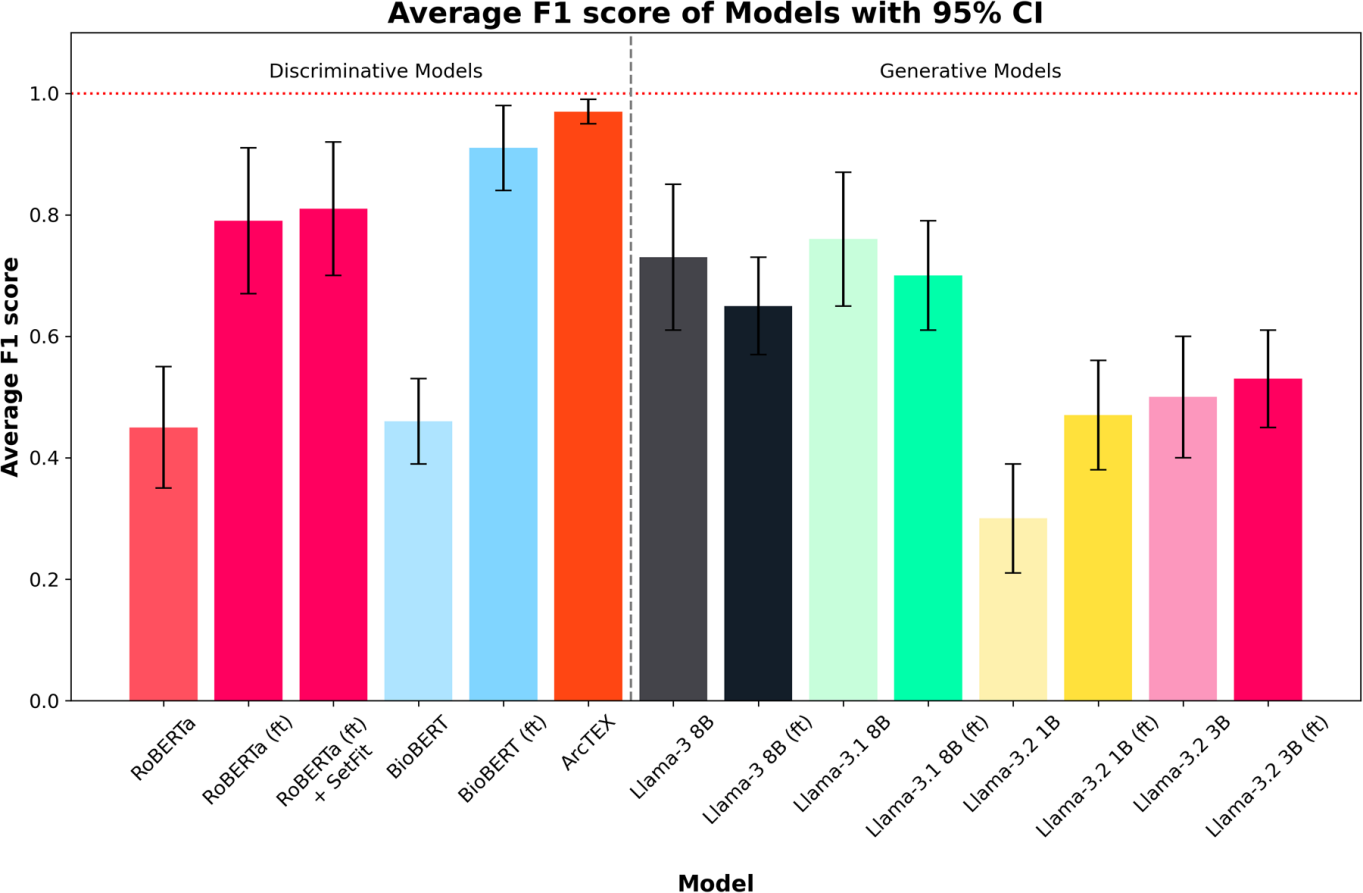
Average F1 scores for each model across all test sets. Error bars show 95% CI. Finetuned models are marked by (ft). ArcTEX shows the highest performance overall with the 95% CI. Generally, the base GMs perform comparably to better than the baseline DMs, however with finetuning the DMs outperform all GMs. Finetuning negatively influences the Llama 3 and 3.1 models.

The overall results can be found in Table 3, with plots of the accuracies displayed in Figure 4 and the F1-scores in Figure 5. The results show clear competition between the base models regardless of type. Generally, the larger base GMs outperform the base DMs, with an average accuracy of 79.83% for Llama 3 and 78.94% for Llama 3.1, compared to ∼64% for RoBERTa and BioBERT. Llama 3.2 (3B) is more comparable to the base DMs with an accuracy of 57.39%, with Llama 3.2 (1B) only managing to achieve 37.56%. Comparatively, the finetuned models follow a different trend. The finetuned DMs in this case perform better than the finetuned GMs, with BioBERT (ft) and RoBERTa (ft) scoring 96.61% and 89.78% accuracy respectively. With the SetFit classifier, a moderate increase in accuracy of 1.16% to 90.94% was observed for RoBERTa (ft). The Llama 3.1 model showed a moderate improvement in accuracy with fine tuning, increasing from 78.94%to 83.33% accuracy and a moderate narrowing in the 95% CI, reflecting greater stability across markers compared to the base model. Llama 3 saw a minor decrease in accuracy, going from 79.83% to 79.22% accurate. This was, however, accompanied by a decrease in the 95% CI width which indicates greater consistency across different clinical features. Both Llama 3.2 models saw an increase in accuracy and a decrease in 95% CI, with Llama 3.2 (1B) scoring higher than Llama 3.2 (3B) with a 95% CI. ArcTEX (a finetuned BioBERT models with SetFit classifier) performed best overall (98.67% accuracy with the lowest 95% CI).

**Table 3:** Accuracies, 95% CI and standard deviations for each of the LLMs tested in experiment 1.

Model		Average Accuracy [%] with 95% CI [LL,UL]	Acc. std dev	Average F1 with 95% CI [LL,UL]	F1 std dev	Average Precision	Average Recall	Average Time per test set [seconds]	Total Time over all test sets [seconds]
Discriminative Models	ArcTEX	**98.67** **[98.13, 99.21]**	**1** **.** **08**	**0.97** **[0.95, 0.99]**	**0** **.** **09**	**0** **.** **97**	**0** **.** **97**	6.36 (122.1)*	114.46 (2,197.73)*
	RoBERTa	64.56 [59.18, 69.94]	10.81	0.45 [0.35, 0.55]	0.21	0.55	0.45	6.01	108.11
	RoBERTa (ft)	89.78 [83.86, 95.7]	11.9	0.79 [0.55, 0.91]	0.24	0.81	0.8	5.94	106.87
	RoBERTa (ft) + SetFit	90.94 [86.0, 95.88]	9.93	0.81 [0.70, 0.92]	0.22	0.84	0.80	**5** **.** **23**	**94** **.** **09**
	BioBERT	64.5 [60.27, 68.73]	8.51	0.46 [0.3, 0.53]	0.16	0.49	0.48	6.65	119.62
	BioBERT (ft)	96.61 [93.86, 99.36]	5.53	0.91 [0.76, 0.98]	0.15	0.92	0.92	6.39	114.93
Generative Models	Llama-3 8B	79.83 [70.34, 89.32]	19	0.73 [0.61, 0.85]	0.24	0.75	0.74	143.4	2,581.23
	Llama-3 8B (ft)	79.22 [73.37, 85.07]	11.77	0.69 [0.53, 0.79]	0.19	0.67	0.69	177.51	3,195.24
	Llama-3.1 8B	78.94 [69.56, 88.32]	18.87	0.8 [0.64, 0.87]	0.21	0.78	0.81	142.15	2,558.75
	Llama-3.1 8B (ft)	83.33 [77.84, 88.82]	11.04	0.75 [0.61, 0.79]	0.14	0.75	0.71	145.11	2,611.96
	Llama-3.2 1B	37.56 [29.83, 45.29]	15.54	0.35 [0.21, 0.39]	0.09	0.35	0.35	54.91	988.35
	Llama-3.2 1B (ft)	64.78 [60.17, 69.39]	9.28	0.47 [0.3, 0.56]	0.18	0.6	0.49	56.95	1,025.09
	Llama-3.2 3B	57.39 [47.64, 67.14]	12.91	0.58 [0.4, 0.64]	0.22	0.59	0.58	82.24	1,480.32
	Llama-3.2 3B (ft)	64.44 [58.31, 70.57]	12.32	0.53 [0.45, 0.61]	0.17	0.59	0.57	106.23	1,912.19

Note for the ArcTEX model, the average and total times reported are for time with pretrained SetFit models, and time including SetFit model training in brackets.

Bold text indicates best results (highest for accuracy, F1, precision, and recall metrics, lowest for time metrics).

*The time difference between running the pipeline with pre-trained setfit models, vs. the time taken to run the model without pre-training of the setit model (in brackets).

The F1-scores follow a different pattern to the accuracies, with the base versions of Llama 3.2 (3B), 3, and 3.1 all outperforming RoBERTa and BioBERT, but with Llama 3.2 (1B) still performing relatively poorly in comparison to the other base models. For the DMs, the F1-score for BioBERT base is higher than that of RoBERTa, with a much smaller 95% CI. Finetuning of the models yields higher average F1-score for the DMs than the GMs, with both RoBERTa (ft) and BioBERT (ft) outperforming Llama 3 and 3.1. The addition of the SetFit classifier for RoBERTa only showed a minor improvement to F1-score and 95% CI range. The F1-scores for the larger GMs are seen to drop compared to the non-finetuned versions. Comparatively, the two Llama 3.2 models increased their F1-scores, reducing the 95% CI in both cases. The ArcTEX model, however, remained the top performer with the highest F1-scores and narrowest 95% CI. As with the accuracy, the ArcTEX model outperformed all others.

The differences between the base and finetuned model F1-scores can be attributed to an imbalance between classes in the test set. Tables 4, 5 demonstrate the difference in misclassification rate between classes. This is caused by the 50:50 split of reports that contain positively identifiable text regarding each marker [for example, “…{feature} stains positively…”, “… is negative for {feature}…”], and reports that either contain no information regarding the given feature or information that cannot be used to determine feature presence [for example, “…staining for (feature) has been requested…”]. Though this would appear as a two-class problem, the positively identified category contains multiple classes ranging from 2 to 21 separate classes depending on the clinical feature. This variability is demonstrated in Table 1.

**Table 4:** Mean number of misclassified answers for each model.

Model	Mean number of misclassified impossible answers	Mean number of misclassified positively identifiable answers	Sum
ArcTEX	**0** **.** **47**	**0** **.** **82**	**1** **.** **29**
BioBERT	7	28.5	35.5
BioBERT (ft)	0.94	2.44	3.38
RoBERTa	1.17	33.28	34.45
RoBERTa (ft)	1.17	9.06	10.23
RoBERTa (ft) + SetFit	1.06	8.0	9.06
Llama-3 8B	12.22	7.94	20.16
Llama-3 8B (ft)	8.67	12.11	20.78
Llama-3.1 8B	13.89	7.17	21.06
Llama-3.1 8B (ft)	2.22	14.44	16.66
Llama-3.2 1B	30.44	32	62.44
Llama-3.2 1B (ft)	2.83	32.39	35.22
Llama-3.2 3B	27.67	14.94	42.61
Llama-3.2 3B (ft)	11.44	24.11	35.55

The number for each row describes how many of the 50 examples in that category were misclassified from the total 100 reports in each test set.

Bold text indicates lowest numbers of misclassified answers for each category.

**Table 5:** Mean number of misclassified results assigned a class group.

Model	Total number of results misclassified using the impossible answer class	Mean number of results misclassified using a positively identified class
ArcTEX	0.22	1.11
BioBERT	13.17	22.33
BioBERT (ft)	0.22	3.17
RoBERTa	24.22	11.22
RoBERTa (ft)	6.56	3.67
RoBERTa (ft) + SetFit	6.56	2.50
Llama-3 8B	1.22	18.94
Llama-3 8B (ft)	4.28	16.50
Llama-3.1 8B	0.83	20.22
Llama-3.1 8B (ft)	8.78	7.89
Llama-3.2 1B	13.22	49.22
Llama-3.2 1B (ft)	23.33	11.89
Llama-3.2 3B	2.28	40.33
Llama-3.2 3B (ft)	9.50	26.06

Note that for the finetuned DMs, both values decrease, but for the finetuned GMs the rate at which misclassified results are assigned an “impossible answer” class increases.

**Figure 6:**
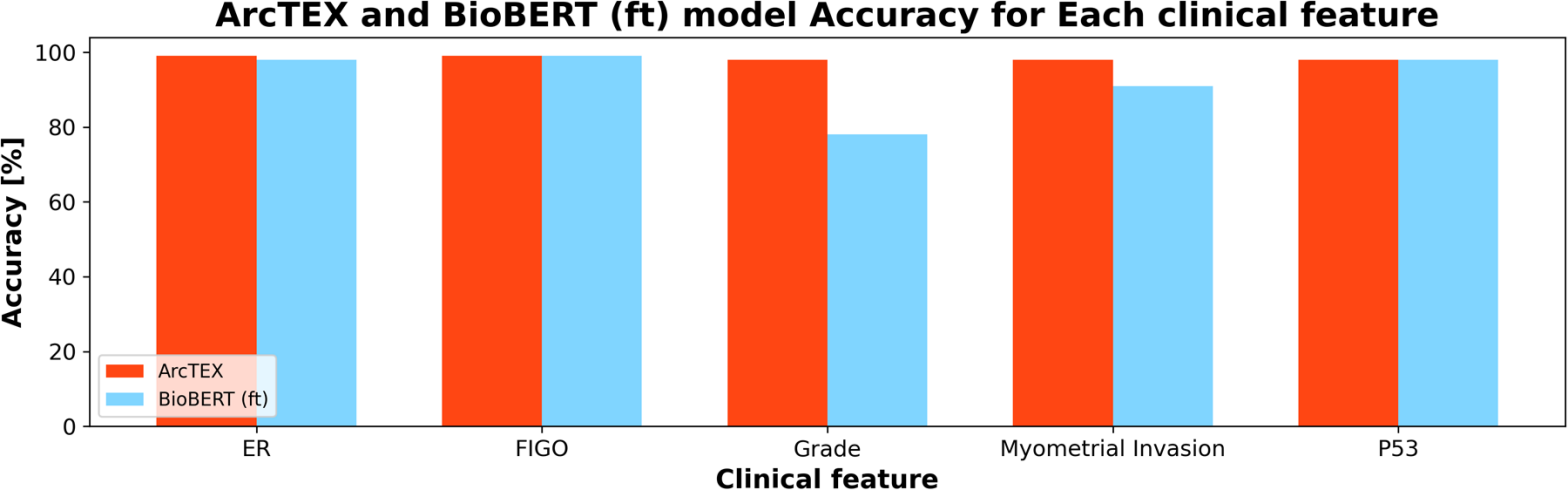
Comparison of ArcTEX and BioBERT finetuned (ft) showing a comparison for a selection of clinical features. Note for some features, BioBERT (ft) struggled in comparison to ArcTEX where in others the results are comparable.

With the DMs, especially for the BioBERT model, the introduction of the SetFit classifier saw an increase in accuracy and F1-scores. Though the QA stage is the same for both models, the ability of the SetFit approach to better classify the outputs into a set of reproducible results outstrips the ability of cosine similarity. This can be demonstrated by comparing two identical QA models with and without the SetFit classifier, such as BioBERT (ft) and ArcTEX. Figure 6 shows how cosine similarity struggles with certain clinical features more than others compared to the SetFit approach. While certain clinical features such as P53 are expressed in clinical notes often similarly as “negative” or “positive”. Other features are documented in a larger variety such as grade, which can be reported as words (i.e., “high”, “low”, “intermediate”) or in numeric values (i.e., “grade 3”, “the grade is 1”, “g3”).

**Figure 7:**
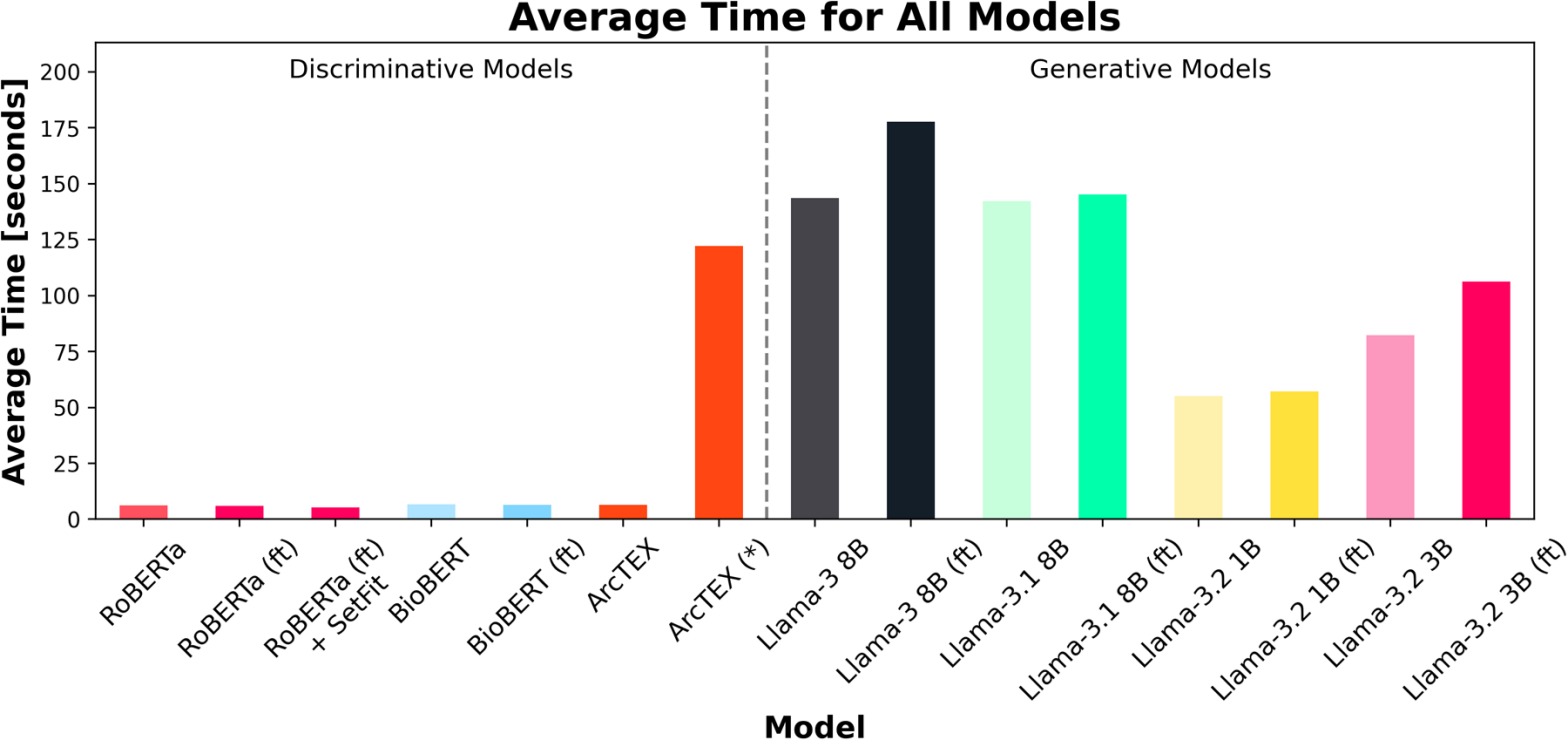
Average time (in seconds) for each of the 18 test sets (100 reports per set) to run per model. Finetuned models are marked by (ft). Note that ArcTEX is plotted twice to demonstrate the difference between run times with and without (denoted by a *) pretrained SetFit models. Note that variation in average time was minimal so has been omitted from plot.

Another feature considered during experiment 1 was the computation time for each model to run inference on the test set. Figure 7 shows the average runtime to compute the results for one clinical feature, consisting of 100 reports. The total runtime for each model can be found in Table 3. As expected, the smaller DMs ran faster than the GMs. In the case of the ArcTEX model and Llama 3.1, the results are presented both with and without a pretrained SetFit model. In principle, a separate SetFit model is trained for each specific clinical feature. If these models have not been previously generated, the time to train each SetFit model will add a significant amount of time to the processing (115.74 s on average). It should be noted that the SetFit models need only be trained once for a given clinical feature and can then be reused regardless of the dataset. In comparison, the runtime of GM's is between 8.59 to 27.78 times higher than the slowest DM [BioBERT (ft)], with a direct correlation between number of parameters and presence of finetuning, and the time taken to run inference.

### 3.3 Experiment 2: adaptability

**Figure 8:**
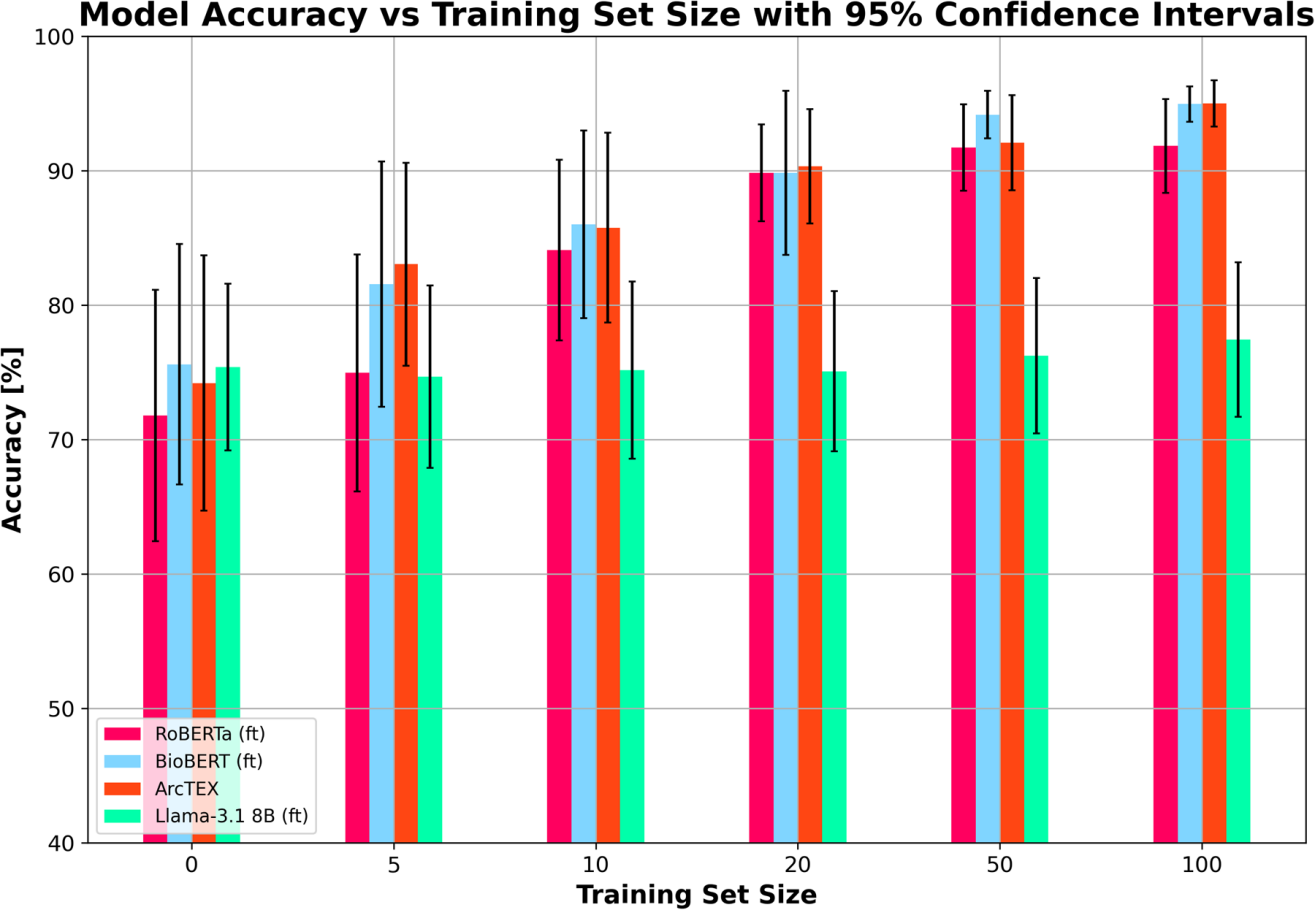
Mean accuracy (and 95% confidence intervals) vs. training set size for finetuned RoBERTa (ft), BioBERT (ft), Llama 3.1 8B (ft) and ArcTEX based on 5 random repetitions with different training data.

The results of experiment 2 can be found in Table 6. Figure 8 shows the average accuracy of ArcTEX, RoBERTa (ft), BioBERT (ft), and Llama 3.1-8B (ft) to extract new oncological features which were not part of the finetuning dataset. The results indicate that the average accuracy for all 4 models is similar [between 71.80% and 75.60% for RoBERTa (ft) and BioBERT (ft) respectively] if no additional feature specific training data is used.

**Table 6:** Results for experiment 2, showing the average accuracy for each training set size over each clinical feature. 95% CI are in square brackets.

Model	Average accuracy with 95% CI [LL, UL]						
	Number of training samples	0	5	10	20	50	100
RoBERTa (ft)		71.8 [62.45, 81.15]	74.96 [66.15, 83.77]	84.08 [77.36, 90.8]	89.84 [86.25, 93.43]	91.72 [88.5, 94.94]	91.84 [88.34, 95.34]
BioBERT (ft)		75.6 [66.67, 84.53]	81.56 [72.44, 90.68]	86 [79.02, 92.98]	89.84 [83.75, 95.93]	94.16 [92.39, 95.93]	94.96 [93.64, 96.28]
ArcTEX		74.2 [64.7, 83.7]	83.04 [75.5, 90.58]	85.76 [78.7, 92.82]	90.32 [86.07, 94.57]	92.08 [88.55, 95.61]	95 [93.29, 96.71]
Llama-3.1 8B (ft)		75.4 [69.2, 81.6]	74.68 [67.9, 81.46]	75.16 [68.58, 81.74]	75.08 [69.11, 81.05]	76.24 [70.47, 82.01]	77.44 [71.69, 83.19]

**Figure 9:**
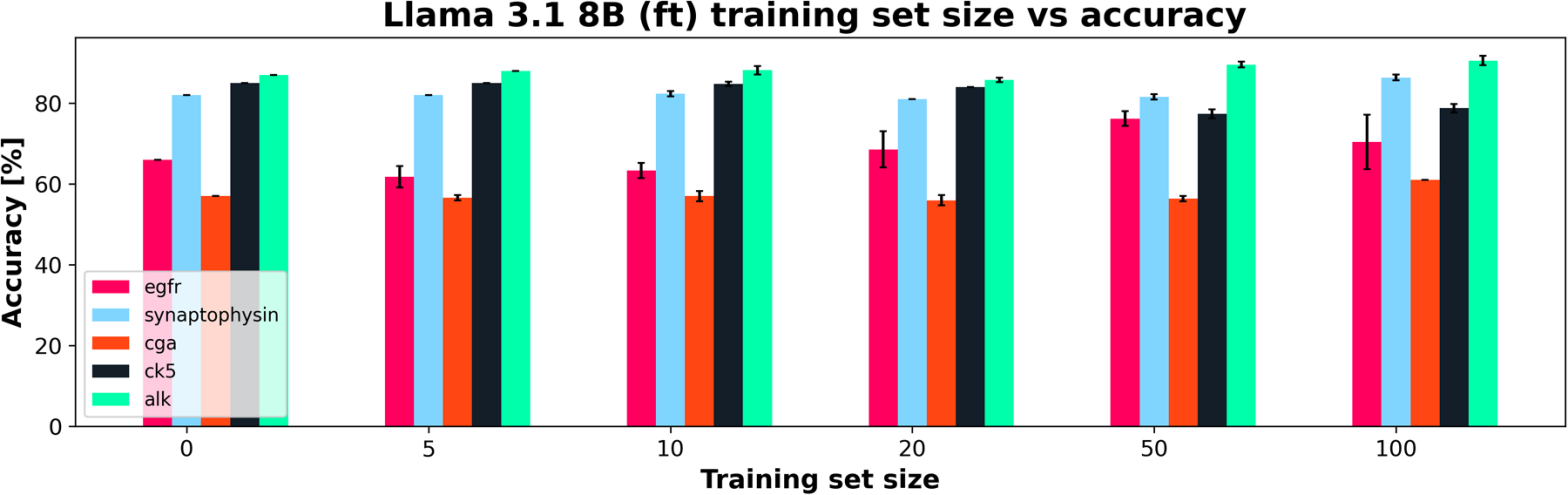
Training set size vs. Accuracy for a finetuned Llama 3.1 8B (ft) showing how each clinical feature's accuracy changed with increasing training set size. 95% CI shown in error bars.

However, when adding additional training data, the Llama 3.1 model did particularly poorly compared to the DMs it was compared to Figure 8 shows the Llama 3.1 model only marginally improving with increasing training set size. This is in line with the results from experiment 1, showing that the variation in the accuracy over all features was the main aspect that was improved with finetuning compared to the base model. Figure 9 shows the average accuracies and 95% CIs for the 5 clinical features tested for the Llama 3.1 8B (ft) model. Each clinical feature shows either poor improvement or a reduction in accuracy with increasing training set size. This is consistent with experiment one which showed only a minor improvement in accuracy but an overall drop in F1-score.

In contrast, the DMs all showed very similar improvements in accuracy with each increase in training set size regardless of model. The increase in training set size not only correlates with an increase in accuracy, but also with a narrowing of 95% CI, showing greater ability of these DMs to adapt to small training set sizes than the Llama 3.1 model. As with the results in experiment 1, the BioBERT (ft) and ArcTEX share very similar results given that ArcTEX's base model is BioBERT. It was observed that the difference in performance between adding 50 samples and 100 samples began to drop off in general. It is likely that around the 50–100 samples mark is the point at which the training plateaus, depending on clinical feature being evaluated.

## 4 Discussion

Here we will discuss the differences between the performance of ArcTEX compared to other comparable DMs and GMs for the task of data extraction through question answering in the setting of free text reports.

The differences in performance between the DMs and GMs for extracting the values of a given clinical feature are shown in Experiment 1: Model comparison. As demonstrated in Table 3, and in Figures 4, 5, without finetuning, BioBERT and RoBERTa, show moderate accuracy of ∼65% with relatively narrow 95% CIs. The F1-scores are, on the other hand, lower, with wider 95% CIs. These scores, both in terms of accuracy and F1-score, are comparable to the smaller GMs (Llama 3.2 1B and 3B). Despite the size differences between these sets of models, both manage comparable results off the shelf with no prior finetuning or in context prompting. In comparison, the larger base Llama models (Llama 3 and 3.1 8B) show slightly higher accuracies and F1-scores across the test set compared to the smaller GMs and DMs. Unsurprisingly, without finetuning, it is evident that the larger models can handle data extraction from free text pathology reports more efficiently than smaller models. This fits with the scaling laws proposed by Kaplan et al., who suggested that model performance depends strongly on its scale (22).

With finetuning, RoBERTa, BioBERT and the ArcTEX model, show a higher mean accuracy and F1-scores compared to finetuned GMs. This agrees with other publications which have demonstrated that simpler BERT based methods are often superior compared to generative LLMs for classical NLP tasks such as NER or RE (10, 23, 24)). The Llama 3 and 3.1 models, in fact, showed decreased mean F1-scores following finetuning. As demonstrated in Table 4, the misclassification rate does not change equally for all models between each type of class which therefore affects the F1-score more dramatically for some models over others. For the DMs, there is a decrease in misclassification rate following finetuning for both positively identifiable answers and impossible answers, leading to the rise in F1-scores seen in Figure 5. In comparison, the GMs demonstrate a drop in misclassified impossible answers assigned a positively identified value, but an increase in reports with positively identifiable answers assigned the impossible answer class with a similar percentage change.

There are a few reasons that could be contributing to the differences seen in the two model types. One reason for this could be due to the way that the models are trained. Within the training set, approximately 25% of the examples are attributed the impossible answers class. Comparatively, the remaining 75% of the training sets are composed of the positively identifiable classes seen in Table 1. Of these, most (10/18) clinical features have more than 3 positively identifiable classes, making it likely that the impossible answers class become the majority class. Previously, transformer based models have been shown to be adept at handling class imbalance compared to other machine learning methods (25). Despite this, finetuning of GMs has also been demonstrated to increase the risk of overfitting in a question answering setting (26). Because of the distribution in the training data, this may have been a factor in the results we see in experiment 2. This may have been avoided through a highly curated training set but would require significant experimentation to optimize given the variability in reporting within a given class for each feature. An additional factor that may have influenced the difference in F1-scores following finetuning is a combination of the finetuning to handle impossible answers and the way that they were prompted. As shown in Figures 2, 3, the prompt template for the GMs provided an instruction to produce a “NaN” where it was unable to answer the question. Where previously the models may have attempted to produce an answer, at times through hallucination, following finetuning it may be the case that the GMs were more likely to answer with “NaN” rather than a hallucination. As shown in Table 5, following finetuning, the rate at which misclassified answers are misclassified with an impossible answer increases compared to those answered with a positive answer. This preference to abstain from providing an answer following finetuning has been previously demonstrated to negatively affect model performance (27). Comparatively, over the five repetitions used in experiment 2, the ability of both ArcTEX and BioBERT to quickly adapted to extract new clinical features from a different oncology area is shown. The accuracy plateaus around 100 training samples as shown in Figure 8. As half of the training samples are without the specific clinical feature being present, only 50 samples need to be manually annotated by a clinical expert.

Overall, the results demonstrate a superior performance of the proposed ArcTEX pipeline compared to a finetuned BioBERT model. This shows that the choice of postprocessing method can significantly enhance model performance for some of the clinical features, which is also reflected in the strong decrease of the standard deviation and CI of the accuracy (see Table 3, Figure 6). The BioBERT pipeline uses cosine similarity to compare the sentence embeddings of predicted answers to annotated examples, which is a widely used method. In contrast, the SetFit approach consists of a two-step approach: first a sentence transformer is fine-tuned and then a classification head is trained (16). The sentence transformer is finetuned using a contrastive learning regime which is very efficient when only a few examples per class are available (in our case between 2 and 22 examples per class). It must be noted that while the SetFit approach increases accuracy, additional time and compute resources are required to train and store the SetFit models, depending on the selected sentence transformer model. In our case, we used Mpnet-v2, which resulted in an additional training time of 115.74s per biomarker and requires 0.5GB additional disc space per model, which can add storage costs when running the pipeline over all biomarkers.

Due to the size of LLMs, there are discussions around the environmental impact and economic costs of training and running LLMs (28, 29). This resulted in the development of small language models (SLMs) recently (30) such as Llama 3.2 1B and 3B. Even though these models are still significantly larger compared to a standard BERT based model, these models have better chances to be executed in a resource constrained environment such as in a hospital setting. However, the results in experiment 1 demonstrate, that finetuned DMs outperform SLMs, such as Llama 3.2, both in terms of accuracy and F1-scores, as well as run time. Though this experiment was performed using a GPU based instance, an additional benefit to the ArcTEX model, as well as the other DMs is their ability to be run on a simpler CPU set up. DMs have been shown to be able to be run and optimized on CPU only set ups (31) where GMs such as the Llama models here have been demonstrated to require GPUs to run (28). This makes DMs ideal for deployment into systems used in healthcare settings such as the NHS where availability of GPUs is unlikely and external access to cloud-based systems is restricted.

Alongside the development of SLMs over increasingly large LLMs, domain specific model development has appeared as a growing frontier (32, 33). Examples such as BioGPT, BioMistral, BioBERT, and ClinicalT5 have been developed in recent years for the biomedical sphere and are pretrained on a corpus of biomedical texts (8). Domain specific models have been shown to outperform larger and more general models in various biomedical tasks previously, especially in zero shot settings (34, 35). Compared to their larger counterparts, DMs such as BERT based models show a greater ability to learn from small training sets or “few-shot” methods, to perform specific information retrieval tasks (36–38). This is further demonstrated by the results shown in experiment 2, with the Llama 3.1 8B (ft) model failing to improve at a rate comparable to the DMs it was compared to.

DMs have been shown previously to be able to more quickly adapt to information retrieval tasks than GMs, and have shown excellent performance against benchmark datasets (39, 40). The results presented here would support that DMs may be better suited to the task of feature extraction than GMs, especially where finetuning dataset size is limited. Because large variations in how individual classes are described for a particular feature, larger datasets are often required to adequately train GMs compared to DMs. Because GMs are developed to provide plausible responses to prompts, it can be difficult to determine whether the generated response is factual or a hallucination (41). Compared with DMs that will return strings from the queried text. Because of these inherent differences, categorisation of outputs becomes much easier to standardise and trust when using DMs over GMs. Various methods can be used to help reduce the number of erroneously generated answers or “hallucinations”, such as utilizing prompt engineering techniques, however the risk of hallucinations can never be fully mitigated (42). Where GMs may excel over DMs in this setting is for complex scenarios where feature extraction is insufficient to answer the question. GMs have been demonstrated to excel in text summarization (43), and have shown promising results when applied to more complex medical data extraction and summarization, for example where a particular feature may be described over multiple sentences or is inferred from multiple statements. Concerns around their use in a clinical setting, however, have been raised (44, 45).

Another technique that has recently been widely adopted within the NLP field, particularly for knowledge-intensive language tasks, is retrieval-augmented generation (RAG) (46). While RAG has shown promise in scenarios requiring dynamic access to external knowledge, we did not include it in our evaluation due to the fact the annotation guidelines used in this study were relatively straightforward and could be fully supplied to the LLMs within their context windows. This inclusion ensured the models had access to all necessary task-specific instructions at inference time, eliminating the need for document retrieval. Nonetheless, we acknowledge that RAG would be a valuable avenue for future work, particularly in the scenario where ArcTEX is extended to extract the values of a more diverse array of biomarkers that would require access to external clinical knowledge.

At present, our evaluation is limited to a single dataset from one NHS trust. Despite the size of the dataset, there is a high likelihood that there may be varying results when utilising the currently evaluated LLMs against data from other trusts. Pathology reports, as with other free text data, will vary between authors which may influence a model's ability to handle them. In future, we plan to extend our analysis to additional trusts, deploying the ArcTEX model on various datasets within different clinical environments. This will entail not only extending to additional clinical features, but to also explore the use of ArcTEX where multiple biopsies/tumours are reported within a single report. We also hope to explore different report styles, such as imaging reports and clinical notes and show the ability of ArcTEX to handle data from a variety of sources and authors.

LLMs have been demonstrated to be potentially invaluable tools in text extraction from free text reports and can be finetuned to handle very specific domains. Though GMs do show promise in this field, and indeed perform better when used “off the shelf” compared to DMs, they do not exhibit the same ability to adapt to new data as easily as the DMs can. Here we have presented ArcTEX, a DM which has shown higher accuracy, and F1-scores compared to the other evaluated models presented here. In addition to its ability to accurately extract clinical features, ArcTEX can also be run in resource constrained environments. Given the lack of GPUs or access to cloud-based GPU clusters within clinical settings such as in hospitals, ArcTEX could provide a viable solution to extracting clinical features from free-text data within a clinical setting. The addition of the post-processing steps to completely remove the risk of PII exposure or leaking also adds to the suitability of ArcTEX for clinical feature extraction in a clinical setting.

## Data Availability

The data analyzed in this study is subject to the following licenses/restrictions: The data used for the purposes of this paper are provided as part of a contract with our NHS partners. As such the data is not available for public access or for sharing out with the terms of our contractual agreements with our partners. Requests to access these datasets should be directed to info@arcturisdata.com.

## Footnotes

^1^ https://huggingface.co/dmis-lab/biobert-base-cased-v1.1-squad

^2^ https://huggingface.co/sentence-transformers/all-mpnet-base-v2

^3^ https://aws.amazon.com/ec2/instance-types/g6/
